# Predictors of Ischemic Stroke After Acute Coronary Syndrome: A Retrospective Analysis

**DOI:** 10.7759/cureus.68692

**Published:** 2024-09-05

**Authors:** Javaria Riaz, Hasan Shafique, Hanzalah Bin Arshad, Muhammad Shahzaib, Amatul Noor, Nadeem Ahmad, Rana Shahzaib Ali, Haris Ur Rehman, Abdullah Saif Khan, Sanwal Mehmood, Muhammad Hamza Riaz, Aimen Karamat, Sana Liaquat, Tayyab Mumtaz Khan

**Affiliations:** 1 Medicine, Mohi-ud-Din Islamic Medical College, Mirpur, PAK; 2 Internal Medicine, Jinnah Hospital, Lahore, Lahore, PAK; 3 Internal Medicine, Shifa International Hospital Islamabad, Islamabad, PAK; 4 Internal Medicine, Tehsil Headquarter (THQ) Hospital Ahmedpur Sial, Jhang, PAK; 5 Medicine, Mayo Hospital, Lahore, PAK; 6 Cardiology, Allama Iqbal Medical College, Lahore, PAK; 7 Orthopaedic Surgery, Sheikh Zayed Medical College and Hospital, Rahim Yar Khan, PAK; 8 Internal Medicine, Rahbar Medical and Dental College, Lahore, PAK; 9 Internal Medicine, Allama Iqbal Medical College, Lahore, PAK; 10 Internal Medicine, Punjab Rangers Teaching Hospital, Lahore, PAK; 11 Orthopaedics, Sheikh Zayed Medical College and Hospital, Rahim Yar Khan, PAK; 12 Orthopaedic Surgery, Rawalpindi Medical University, Rawalpindi, PAK

**Keywords:** acute coronary syndrome, ischemic, predictors, retrospective, stroke

## Abstract

Background

Ischemic stroke (IS) is a fatal complication of acute coronary syndrome (ACS). Factors that speed up IS development after ACS are understudied, especially in developing countries like Pakistan. Thus, this study was designed to identify the potential risk factors of IS in patients with a preceding episode of ACS.

Methodology

This retrospective study was performed on 208 patients whose ACS and its consequent complications such as IS were managed in the cardiac and neurology units of Benazir Bhutto Hospital, Rawalpindi, from January 2022 to March 2023. Patients were enrolled via consecutive sampling and pre-defined inclusion and exclusion criteria. Before data collection, informed consent and ethical approval were obtained. Data were retrieved from the medical records of the patients. A self-structured proforma was applied to collect data. SPSS version 25 (IBM Corp., Armonk, NY, USA) was used for data analysis. The study variables between patients with and without IS were compared using descriptive and inferential statistics. The association between IS and its possible risk factors in patients who had previously experienced ACS was determined using multivariate logistic regression.

Results

Of the 208 enrolled patients, 24 (11.54%) had IS following ACS. Sedentary lifestyle (odds ratio (OR) = 3.099, 95% confidence interval (CI) = 1.025~4.219, p = 0.009), hypertension (OR = 3.060, 95% CI = 1.798~4.876, p = 0.002), diabetes mellitus (OR = 2.899, 95% CI = 1.126~4.112, p = 0.009), dyslipidemia (OR = 2.907, 95% CI = 1.332~4.254, p = 0.007), history of smoking (OR = 2.760, 95% CI = 1.234~4.122, p = 0.018), and non-adherence to ACS medication (OR = 2.966, 95% CI = 1.300~4.266, p = 0.030), were the risk factors of IS among patients with preceding ACS.

Conclusions

In the study population, the incidence of IS following ACS was significant. Sedentary lifestyle, hypertension, diabetes mellitus, dyslipidemia, smoking history, and non-compliance with ACS therapy all played a significant role in the development of IS in patients with prior ACS. Proper management of ACS and associated risk factors could lead to the prevention of serious complications such as IS.

## Introduction

Stroke is a brain disease that causes blockage of the brain’s major arteries, reduces oxygen supply to the brain, and, ultimately, results in death or disability. There are various types of stroke, such as transient ischemic attack, hemorrhagic stroke, and ischemic stroke (IS) [1]. Globally, stroke has emerged as the second leading cause of death and morbidity after ischemic heart disease [2]. It is estimated that approximately 15 million people develop stroke annually worldwide. In the last three decades, the prevalence of stroke in developing countries has increased at an annual rate of 14.30%. Stroke is responsible for about 67.30% of deaths related to the brain [3].

IS is a fatal consequence of acute coronary syndrome (ACS) [4]. ACS is characterized by reduced blood supply to the heart due to the narrowing of the heart’s coronary vessels secondary to plaque rupture or erosion in the vessel that causes total or partial blockage in the vessels. ACS has three subtypes including ST-elevation myocardial infarction (STEMI), non-ST-elevation myocardial infarction (NSTEMI), and unstable angina (UA) [2,5]. According to previous observational data, the incidence of stroke after ACS ranges between 0.5% and 2.1% during hospitalization and between 0.7% and 2.1% within 30 days of ACS. The cumulative incidence of IS after ACS could reach up to 21.40% in a few years after ACS [6-8].

Both ACS and IS have common risk factors such as diabetes mellitus, hypertension, lack of physical activity, dyslipidemia, and smoking. ACS and IS have similar pathogenesis, including inflammation and atherosclerosis. ACS can increase the risk of stroke owing to thromboembolism, ischemia-induced atrial fibrillation (AF), or blood stasis [9,10]. Although anticoagulation therapies are recommended after ACS-induced AF and atrial flutter, as these conditions are associated with a significantly increased risk of cardioembolic stroke, no risk assessment system has been developed to identify the patients at high risk for IS following ACS [11-16].

In the absence of an established scoring system for the assessment of the risk of IS after ACS, the determination of the predictors of IS among ACS patients could help in controlling the morbidity and mortality related to IS after ACS. Thus, the objective of this study is to determine the possible risk factors of IS in patients with prior ACS.

## Materials and methods

Study design and study population

We conducted this retrospective cohort study in the cardiology and neurology units of Benazir Bhutto Hospital (BBH), Rawalpindi, Pakistan. Data were gathered from medical records of 208 patients who received treatment at BBH for ACS and its complications between January 2022 and March. The WHO calculator was used for sample size calculation, and patients were recruited in the study via the consecutive sampling technique.

Inclusion and exclusion criteria

All patients of either gender, aged 40 years or above, with complete medical records of ACS (ACS was diagnosed via electrocardiogram (ECG), cardiac biomarkers, and coronary angiography for accuracy), and its consequent complications management in BBH were included in the study. Patients with incomplete medical records, a history of stable angina, hemorrhagic stroke, congenital heart disease, severe liver disease, autoimmune disease, active infectious disease, and oncological conditions were excluded from the study.

Acute coronary syndrome and ischemic stroke diagnosis

ACS subtypes were defined according to the American Heart Association. STEMI was described as chest discomfort together with ST-segment elevations (new ST-segment elevation at the J point in at least two contiguous leads of 2 mm (0.2 mV) or more in men and 1.5 mm (0.15 mV) in women in leads V2-V3 or 1 mm (0.1 mV) in other contiguous leads or the limb leads) or new-onset left bundle branch block on ECG and increased cardiac biomarkers (troponin I). NSTEMI was defined as chest discomfort associated with raised cardiac biomarkers of myocardial ischemia and no ST-segment elevation instead of ST-segment depression at the J point of 0.5 mm (0.05 mV) or above in two or more contiguous leads or new-onset T-wave inversion on ECG. UA was described as anginal pain that occurs at rest but does not have ST-segment elevation or increased biomarkers of myocardial ischemia [2,5]. Stroke was described as brain dysfunction produced by an ischemic or hemorrhagic event with remaining symptoms at least 24 hours after onset or resulting in death [1].

Ethical approval

Ethical approval was obtained from BHB’s Ethical Review Board, Rawalpindi, Pakistan (approval number: BBH.ERB.283/101). After properly explaining the study, informed consent was obtained from each participant during their follow-up visit at BBH.

Data collection

The medical records of patients comprised detailed history, physical examination findings, and investigation reports. Investigations including CT scan of the brain, ECG, cardiac biomarkers (troponin I; normal = <0.04 ng/mL, abnormal = >0.04 ng/mL), coronary angiography, glycated hemoglobin (HbA1c) levels (normal = <5.7%, abnormal = >5.7%), hemoglobin level (for men: normal = 13.2-16.6 g/dL, abnormal = <13.2 g/dL; for women: normal = 11.6 to 15 g/dL, abnormal = <11.6 g/dL), and serum lipid/cholesterol level (normal = <200 mg/dL, abnormal = >200 mg/dL) were performed during their management at BBH. All data were retrieved from the medical records of the patients. A self-designed proforma was applied for data collection. It had two components. The first included history and examination findings such as age, gender (male or female), lifestyles (sedentary lifestyle: sitting or lying for six or more hours per day or daily walk of less than 15 minutes, active lifestyle: sitting or lying for less than six hours per day or daily walk of 15 or more minutes), body mass index, ACS subtype (UA, STEMI, or NSTEMI), non-adherence to ACS medication (yes or no), history of any revascularization procedure after ACS that was done previously (yes or no), and presence or absence of comorbidities (diabetes mellitus, hypertension, anemia, dyslipidemia, and smoking history). The investigation reports were noted in the second component. Weight was divided by height squared to determine the body mass index (BMI).

Data analysis

The statistical analysis in this study was conducted using SPSS Statistics for Windows, Version 25 (IBM Corp., Armonk, NY, United States). Quantitative data were shown through mean ± standard deviation (SD), whereas qualitative data were presented via frequencies and percentages. To compare the groups for numerical and nominal variables, independent samples t-test and chi-square test were applied, respectively. Predictors of IS were assessed in the study via a multivariate logistic regression model. P-values <0.0 5 were considered significant.

## Results

The prevalence of IS among patients after one episode of ACS was 11.54% (n = 24). The means of study variables such as age and BMI were 63.50 years with an SD of ±12.23 and 24.06 kg/m^2^ with an SD of ±6.12, respectively. Table 1 presents the characteristics of the study population. The incidence of IS was higher among older male patients who had a sedentary lifestyle, higher BMI, NSTEMI subtype of ACS, hypertension, diabetes mellitus, anemia, dyslipidemia, history of smoking, non-adherence with ACS medications, and no revascularization procedure after ACS. A statistically significant difference was noted in the lifestyles, hypertension, diabetes mellitus, dyslipidemia, history of smoking, and adherence to ACS medication with a p-value <0.05 for all these risk factors between the IS group and the non-IS group. No significant differences were noted between the IS and non-IS groups in age, gender, BMI, ACS subtypes, anemia, previous history of stroke, and history of revascularization procedure with a p-value >0.05 for all these variables.

**Table 1:** Demographic characteristics of the study population and the chi-square test findings of the study variables. BMI = body mass index; ACS = acute coronary syndrome; UA = unstable angina; NSTEMI = non-ST-segment elevation myocardial infarction; STEMI = ST-segment elevation myocardial infarction

Variables of ACS patients (N = 208)	Status	Ischemic stroke group (n = 24)	Non-ischemic stroke group (n = 184)	Independent t-test/Chi-square test
Age (years)		65.12 ± 12	62.14 ± 15	0.08
Gender	Male	14 (58.33%)	115 (62.50%)	0.05
	Female	10 (41.67%)	69 (37.50%)	
Lifestyle	Sedentary	18 (75.00%)	106 (57.60%)	0.008
	Active	6 (25.00%)	78 (42.40%)	
BMI (kg/m^2^)		25.89 ± 6.08	22.23 ± 5.90	0.07
ACS subtypes	UA	3 (12.50%)	45 (24.50%)	0.06
	NSTEMI	14 (58.34%)	75 (40.75%)	
	STEMI	7 (29.16%)	64 (34.75%)	
Hypertension	Yes	19 (79.16%)	131 (71.20%)	0.003
	No	5 (20.84%)	53 (28.80%)	
Diabetes mellitus	Yes	17 (70.83%)	116 (63.10%)	0.007
	No	7 (29.17%)	68 (36.90%)	
Anemia	Yes	13 (54.16%)	93 (50.54%)	0.080
	No	11 (45.84%)	91 (49.46%)	
Dyslipidemia	Yes	17 (70.83%)	118 (64.13%)	0.008
	No	7 (29.17%)	66 (35.87%)	
History of smoking	Yes	16 (66.68%)	108 (58.69%)	0.016
	No	8 (33.32%)	76 (41.31%)	
Non-adherence to ACS medication	Yes	19 (79.16%)	120 (65.22%)	0.025
	No	5 (20.84%)	64 (34.78%)	
History of revascularization for ACS	Yes	10 (41.67%)	70 (38.10%)	0.089
	No	14 (58.33%)	114 (61.90%)	

Table 2 indicates the variable allocation in the multivariate logistic regression analysis. Potential risk factors that showed significant differences in the univariate analysis were put into multivariate logistic regression for additional assessment.

**Table 2:** Allocation of variables for multivariate logistic regression. ACS = acute coronary syndrome.

Variables	Allotment	Variables
Ischemic stroke	Y	1 = yes, 2 = no
Lifestyle	X1	1 = sedentary, 2 = active
Hypertension	X2	1 = yes, 2 = no
Diabetes mellitus	X3	1 = yes, 2 = no
History of smoking	X4	1 = yes, 2 = no
Dyslipidemia	X5	1 = yes, 2 = no
Non-adherence to ACS medication	X6	1 = yes, 2 = no

Logistic regression analyses showed that sedentary lifestyle (odds ratio (OR) = 3.099, 95% confidence interval (CI) = 1.025~4.219, p = 0.009), hypertension (OR = 3.060, 95% CI = 1.798~4.876, p = 0.002), diabetes mellitus (OR = 2.899, 95% CI = 1.126~4.112, p = 0.009), dyslipidemia (OR = 2.907, 95% CI = 1.332~4.254, p = 0.007), history of smoking (OR = 2.760, 95% CI = 1.234~4.122, p = 0.018), and non-adherence to ACS medication (OR = 2.966, 95% CI = 1.300~4.266, p = 0.030) were the predictors of IS among patients after one episode of ACS (Table 3).

**Table 3:** Determination of predictors of ischemic stroke after acute coronary syndrome (ACS) via logistic regression analysis. OR = odds ratio; Cl = confidence interval

Variables	Beta coefficient	OR	95% CI	P-value
Lifestyle	0.176	3.099	1.025~4.219	0.009
Hypertension	0.170	3.060	1.798~4.876	0.002
Diabetes mellitus	0.156	2.899	1.126~4.112	0.009
Dyslipidemia	0.160	2.907	1.332~4.254	0.007
History of smoking	0.151	2.760	1.234~4.122	0.018
Non-adherence to ACS medication	0.159	2.966	1.300~4.266	0.030

## Discussion

The risk of serious complications in ACS patients increases in the presence of comorbidities. One of the most significant and common complications in patients with preceding ACS is IS. Given the notable incidence of IS in patients with prior ACS, determining the potential risk factors that accelerate the development of IS after one episode of ACS in these patients could assist physicians in preventing IS by recognizing the most vulnerable patients. This study has highlighted the incidence of IS and its predictors in previously diagnosed cases of ACS.

In this study, the incidence of IS in patients after one episode of ACS was 11.54% (n = 24). A study from Finland reported a relatively lower incidence of IS at 9.0% [10]. A meta-analysis documented the incidence of IS after ACS up to 21.40% [6]. These variations in the percentages of IS in patients following ACS in different parts of the world could be due to disparity in the prevalence of distinctive possible risk factors of IS and differences in the effectiveness of management of prior ACS which itself is a risk factor for IS. Moreover, this difference could be actual differences in the epidemiology of stroke or indicate the heterogeneity in the methodology of the studies.

The results of this study have also shown that a sedentary lifestyle, hypertension, diabetes mellitus, dyslipidemia, history of smoking, and non-compliance with the ACS medication were significant predictors of IS among patients with prior ACS.

A sedentary lifestyle was associated with the increased incidence of IS in ACS patients. Several studies have endorsed this finding of the current study about the correlation between lack of activity and IS [1,14]. Lack of exercise could lead to obesity and overweight which are conditions directly linked to adverse cardiovascular events.

In this study, hypertension and diabetes were also identified as independent risk factors for IS after ACS. Similar roles of hypertension and diabetes have been documented in different studies in the literature [7,11]. Hypertension and diabetes mellitus both cause cardiovascular ischemic events by inducing atherosclerosis and arteriosclerosis in vessels. These changes in vessels cause turbulence in blood flow, plaque formation, and plaque rupture, eventually resulting in thrombus formation and ischemic events, especially in the heart and brain [15].

Another important determinant of IS in this study population was dyslipidemia. Patients with dyslipidemia had a higher risk of stroke than patients without dyslipidemia. Dyslipidemia could lead to stroke and cardiac incidents as cholesterol influences blood circulation directly. In the literature, several studies have demonstrated similar results regarding the association between stroke and dyslipidemia [2,13].

Smoking was also found to raise the incidence of IS after ACS in the current study participants. Smoking doubles the risk of IS by increasing blood pressure and reducing the blood oxygen level. Another study has also presented smoking as an important risk factor for adverse cardiovascular events after ACS [16].

This study also demonstrated that patients who were non-compliant with prescribed medications were more likely to develop a stroke in contrast to patients who were compliant with recommended therapy. A similar impact of non-adherence to prescribed drugs was also noted in two different studies [17,18].

As we noted, stroke is multifactorial, and patients, especially those who are at risk, should be mindful of their level of exposure and avoid those predisposing factors. Without any doubt, ACS itself plays a role in the development of IS; however, the risk of IS further increases by other modifiable factors such as inactive lifestyle, hypertension, diabetes mellitus, dyslipidemia, smoking, and non-adherence to medications. Therefore, medical professionals must guide patients with ACS in managing their condition, which includes adherence to prescribed medication, adequate exercise, and control of modifiable risk factors.

Limitations of the study

This study is one of the first to highlight the incidence of IS and its potential risk factors in patients with preceding ACS. However, this study also has several limitations. First, this was a single-center study which raises the possibility of bias. Second, this study did not present the causal association between stroke and its risk factors in ACS patients because of its study design. Third, this study did not elaborate on the role of diet in the development of stroke. Fourth, because of the small sample size of the study, its results cannot be generalized. To prevent any bias, we encourage other researchers to conduct future studies at multiple locations with different study designs, large sample sizes, and the inclusion of diet’s role in the development of stroke.

## Conclusions

In this study, the incidence of IS with a preceding ACS episode in the study population was significant. The incidence of IS after ACS was higher among participants of older age and male gender. Moreover, a sedentary lifestyle, hypertension, diabetes mellitus, dyslipidemia, history of smoking, and non-adherence to ACS medications were the significant risk factors for IS development following an episode of ACS in the study population. Therefore, this study suggests that it is mandatory to manage patients with ACS adequately, particularly those who have one or more predisposing factors of IS after ACS. Early and aggressive management of the relevant risk factor of IS after ACS could assist in the prevention of fatal complications of ACS. Health authorities should also educate people about the risk factors of IS in patients with ACS and the importance of compliance with the recommended treatment for ACS.

## Appendices

**Table 4:** The self-designed questionnaire. Lifestyles: sedentary lifestyle = sitting or lying for six or more hours per day or a daily walk of fewer than 15 minutes; active lifestyle = sitting or lying for less than six hours per day or a daily walk of 15 or more minutes. Cardiac biomarker: troponin I: normal = less than 0.04 ng/mL, abnormal = 0.04 ng/mL or above. Glycated hemoglobin (HbA1c) level: normal = below 5.7%, abnormal = 5.7% or above. Hemoglobin level: for men: normal = 13.2 to 16.6 g/dL, anemia/abnormal = less than 13.2 g/dL; for women: normal = 11.6 to 15 g/dL, anemia/abnormal = less than 11.6 g/dL. Serum lipid/cholesterol level: normal = less than 200 mg/dL, abnormal = above 200 mg/dL.

Self-designed questionnaire for the predictors of ischemic stroke after acute coronary syndrome					
First component (regarding the history and physical examination)					
Question number	Research questions	Please write the answer or tick the answer			
1	Age	………. (in years)			
2	Gender	Male		Female	
3	Lifestyles	Sedentary		Active	
4	Body mass index	Weight…… (kg), height…… (m), BMI = weight/height^2^………. (kg/m^2^)			
5	Acute coronary syndrome type	Unstable angina	Non-ST-segment elevation		ST-segment elevation
6	Non-adherence to medications	Yes		No	
7	History of revascularization procedure after acute coronary syndrome	Yes		No	
Presence or absence of comorbidities					
8	Diabetes mellitus	Yes		No	
9	Hypertension	Yes		No	
10	Anemia	Yes		No	
11	Dyslipidemia	Yes		No	
12	History of smoking	Yes		No	
Second component (regarding the findings of investigations)					
Question number	Investigations	Findings			
13	Electrocardiogram				
14	Cardiac biomarker (troponin I)	…… (ng/mL)	Normal		Abnormal
15	Coronary angiography				
16	HbA1c level (glycated hemoglobin)	….. (%, percentage)	Normal		Abnormal
17	Hemoglobin level	….. (g/dL)	Normal		Abnormal
18	Serum lipid level (cholesterol)	….. (mg/dL)	Normal		Abnormal
